# Hybrid endoscopic-microscopic surgery for dumbbell-shaped trigeminal schwannoma: case report and literature review

**DOI:** 10.3389/fonc.2023.1137711

**Published:** 2023-05-19

**Authors:** Xinning Li, Song Han, Xiaoyu Sun, Yang Bai, Qiyan Zhang, Sizhe Feng, Guobiao Liang

**Affiliations:** Department of Neurosurgery, General Hospital of Northern Theater Command, Shenyang, Liaoning, China

**Keywords:** dumbbell-shaped trigeminal neurinoma, supracerebellar-infratentorial approach, subtemporal approach, synchronous endoscopy and microsurgery, skull base lesions

## Abstract

**Background:**

The surgery of dumbbell-shaped trigeminal neurinomas (TN) remains one of the most formidable challenges for neurosurgeons because of its location at great depth in the cranium and proximity to vital neurovascular structures.

**Objective:**

To describe the feasibility of a novel technique, synchronous endoscopy and microsurgery *via* combined far-lateral supracerebellar-infratentorial and subtemporal approach, for resection of this rare entity.

**Methods:**

A 53-year-old women presented with progressive left facial numbness for 2 months. Imaging examinations revealed a left-sided dumbbell-shaped TN afflicting the middle and posterior cranial fossa, and a single-stage combined multiportal endoscopic microscopic approach was attempted for tumor resection. Initially, a purely endoscopic far-lateral supracerebellar-infratentorial approach was used to remove the posterior fossa component with the aid of tentorium incision. Subsequently, a microsurgical subtemporal interdural approach was performed for the exposure and separation of tumor within the Meckel cave. Finally, the tumor was pushed into the porus trigeminus under microscopy, thus enabling tumor extraction for the supracerebellar space under endoscopy without anterior petrosectomy.

**Results:**

The patient evolved favorably without additional neurological deficit after surgery, and postoperative imaging showed a complete resection of the tumor.

**Conclusion:**

We describe the first account of multi-corridor hybrid surgery for removal of TN in a dumbbell configuration, which enables one-stage total tumor removal with minimal added morbidity. This hybrid technique may be an effective piece of the surgeon’s armamentarium to improve outcomes of patient with complex skull-base lesions. Further studies with larger case numbers are warranted to confirm the prognostic significance of this technique.

## Introduction

Trigeminal neurinomas (TNs) are overwhelmingly benign neoplasms that constitute only 0.2% of all intracranial tumors. Complete surgical removal is believed to offer the best chance of cure (1). TNs can originate from any part of the trigeminal nerve sheath from the schwann-oligodendroglia junction to the distal extracranial branches, and therefore may grow into single or multiple distinct compartments, including the subdural (cerebellopontine angle), interdural (lateral wall of the cavernous sinus and Meckel cave), and epidural or extracranial (orbit, pterygopalatine fossa, and infratemporal fossa) spaces (2). Among them, dumbbell-shaped TNs constitute the major subtype, classically with an anterior component in the Meckel cave (MC) and a posterior part extending into the posterior fossa through the porus trigeminus (PT) (2, 3). Resection of dumbbell TNs presents neurosurgeons with a special challenge owing to its deep location as well as relation to many critical neurovascular structures. Skull-base approaches predominate current surgical strategies of dumbbell TNs, which basically consist of anterior transpetrosal approach for posterior fossa tumors, a subtemporal interdural approach for middle fossa counterparts, or a combination of them. The retrosigmoid approach might be auxiliary for posterior fossa tumors with large size (2, 4, 5). However, the optimal approach to minimize approach-related morbidity and improve the extent of resection still remains to be explored.

As a distinct variant of the median supracerebellar-infratentorial approach (SCITA), far-lateral SCITA was initially described for accessing the posterolateral mesencephalon (6). Later, neurosurgeons proved its clinical practicality in treating tumors in centrally-located intra-axial structures (including the splenium, pulvinar and mesial temporal lobe) and skull-base extra-axial tumors (e.g., petroclival meningiomas), without the necessity of traditionally transcortical and extensive skull-base approaches (7). In the last few years, the rapid development of neuro-endoscopy has brought a great opportunity for the improvement of this approach. Clinically, the purely endoscopic far-lateral SCITA (EF-SCITA) has been used for removal of supratentorial lesions with the aid of tentorium incision, including temporal lobe cavernous malformation (8), petroclival meningiomas extending into the middle fossa (9), and retroinfundibular craniopharyngioma (10). These successful experience motivated us to use the same technique for TN resection.

In this case, we described a multi-modality, multi-directional approach for removing TN with a dumbbell configuration, with EF-SCITA approach for resection and microsurgical subtemporal interdural approach for assistance. In the discussion, we summarize breakthroughs and insights on operative approaches for dumbbell-shaped TN, and analyze the strengths and weaknesses of each passage from the perspective of surgery.

## Case description

**Figure 1:**
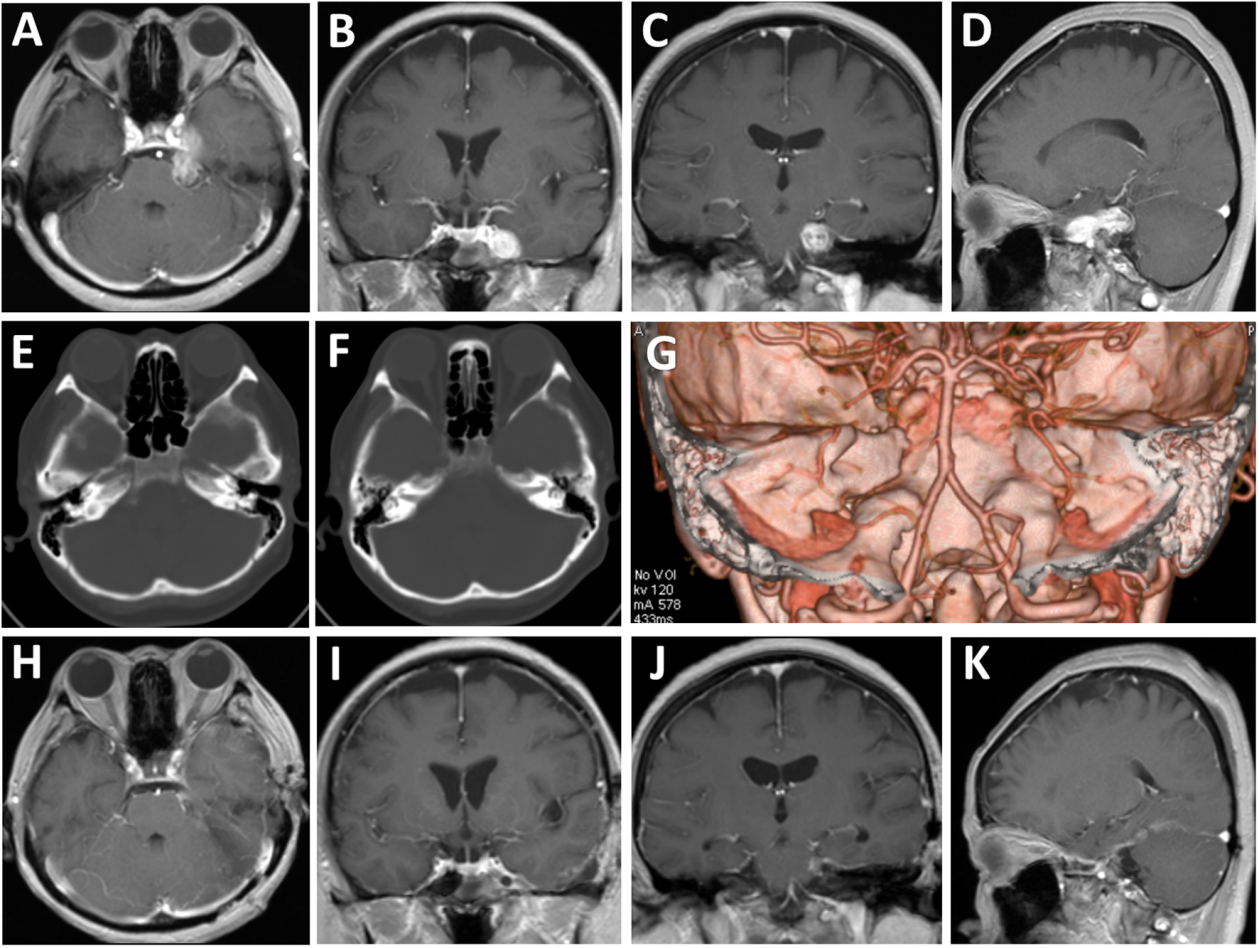
Radiological evaluation of the multi-compartmental lesion preoperatively and postoperatively. **(A–D)** Preoperative axial **(A)**, coronal **(B, C)**, and sagittal **(D)** T1-weighted magnetic resonance images with gadolinium enhancement. **(E–G)** Preoperative tomography images of bone windows **(E, F)** and three-dimensional reconstruction **(G)** revealed no erosion of the middle cranial fossa base and truncation of the petrous apex on the left. **(H–K)** Postoperative axial **(E)**, coronal **(F, G)**, and sagittal **(H)** T1-weighted magnetic resonance images with gadolinium enhancement.

A 53-year-old female presented with progressive left facial numbness and mastication difficulty of two months duration. On examination, she exhibited decreased sensation to light touch over the left V1 to V3 distribution, with no other cranial nerve associated symptoms or signs. Magnetic resonance images showed a well-circumscribed mass in the para-cavernous sinus area with extension into the petroclival region. The dumbbell-shaped lesion appeared hypointense signals on T1-weighted images and hyper-intense on T2-weighted images, with remarkable enhancement by gadolinium. Compression of the left temporal lobe and brainstem was present (**Figures 1A–D**). Computed tomographic scans revealed no erosion of the middle cranial fossa base and truncation of the petrous apex on the left (**Figures 1E–G**). These clinical and radiological findings suggested a diagnosis of left Jefferson type-C TN afflicting the middle and posterior cranial fossa.

**Figure 2:**
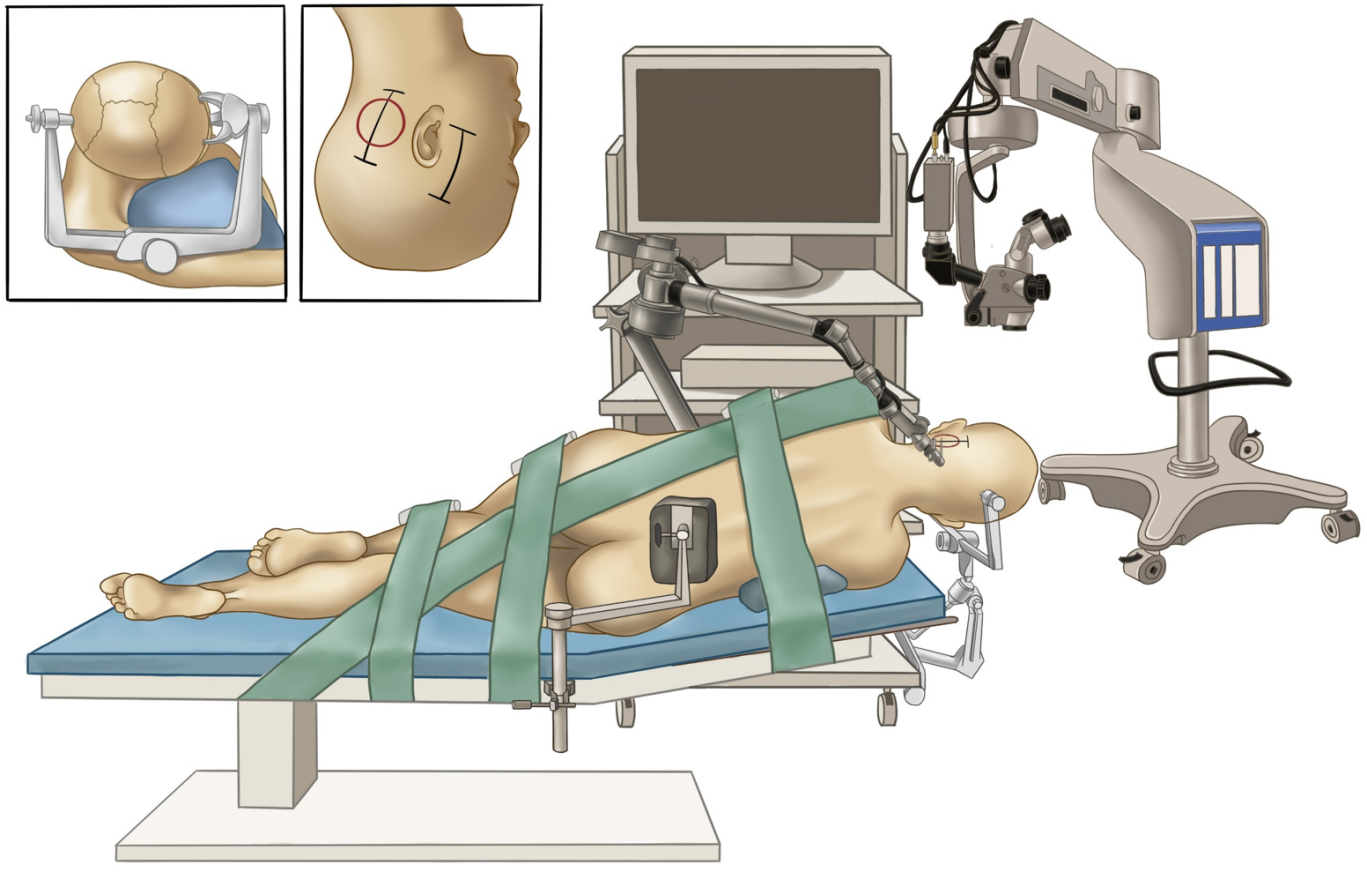
Surgical setup for the hybrid operation. The painting shows the layout of our operating room and the lateral positioning of the patient. The inset figures indicate head positioning (left) and skin incisions (right).

A lumbar drain was placed pre-operatively for intraoperative drainage to reduce brain retraction and obtain as wide an operative view as possible. Then, the patient was placed in right lateral position with the upper body elevated 10°. Unlike the position described before (9, 10), the head was placed with no rotation for maintaining the ear at the top to guarantee synchronous microsurgical and endoscopic operations (**Video**). The endoscope monitor (Karl Storz) was placed in front of the patient, with the pneumatic arm holder placed on the contralateral bedside, while the microscope was placed at the head end (**Figure 2**). Physiological neuromonitoring was also used during surgery to avoid neural complications.

**Figure 3:**
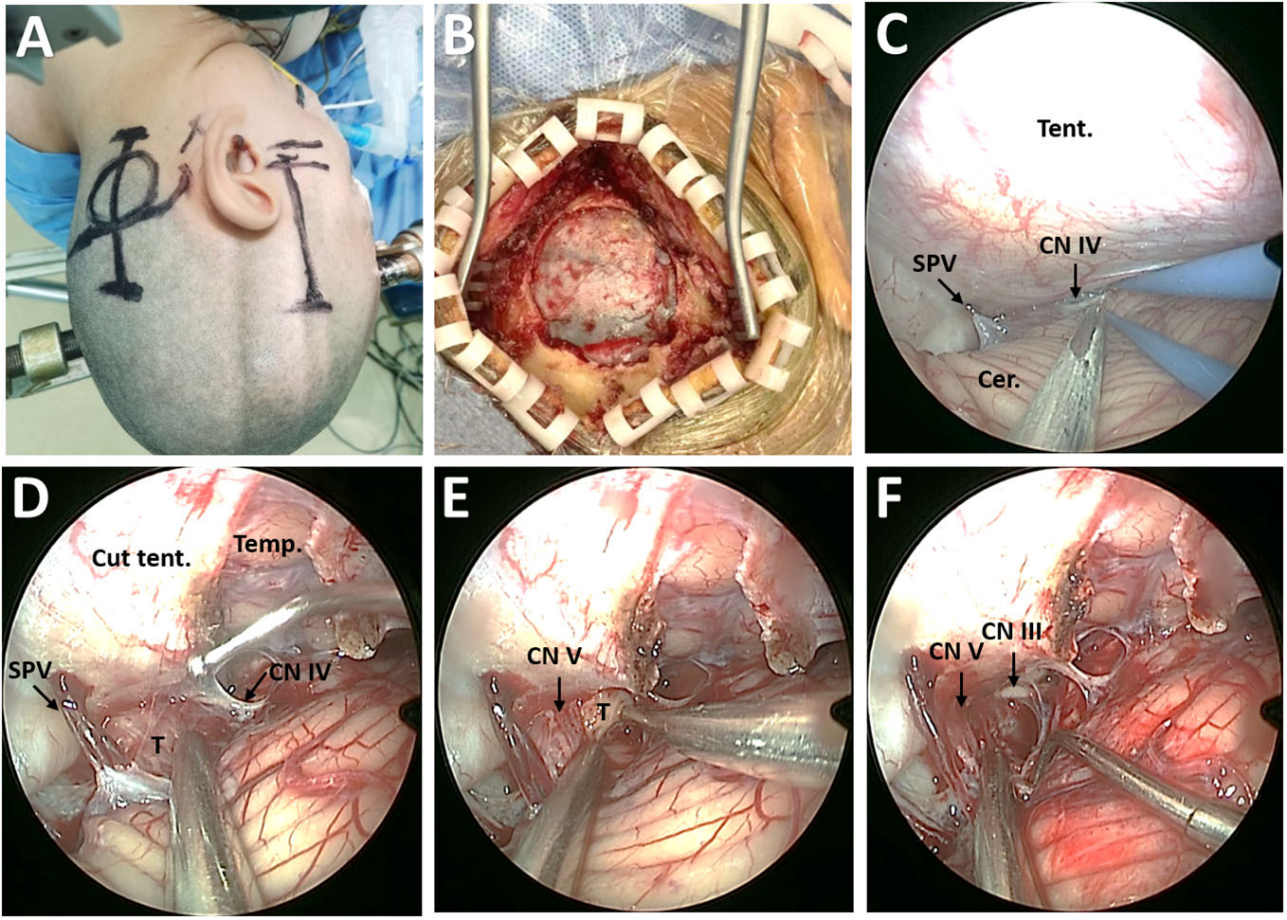
Surgical procedure of EF-SCITA approach for resection of posterior fossa tumor. **(A)** Skin incision for lateral suboccipital craniotomy (left) and subtemporal craniotomy (right). **(B)** Bone window for exposure of the transverse sinus and the inner edge of the sigmoid sinus. **(C)** Endoscopic explorations in the lateral supracerebellar-infratentorial space. **(D)** The exposure of posterior fossa tumor in the petroclincal region after tentorium incision. **(E)** Tumor resection in a piecemeal fashion. **(F)** The exposure of CN V and CN III after tumor resection. Cer., cerebellum; CN III, oculomotor nerve; CN IV, trochlear nerve; CN V, trigeminal nerve; SPV, superior petrosal vein; T, tumor; Temp., temporal lobe; Tent., tentorium cerebelli.

A small straight retroauricular incision was first performed to obtain exposure of the lateral suboccipital region (**Figure 3A**). Then, the entire transverse sinus and the proximal part of the sigmoid sinus were revealed through suboccipital craniotomy (**Figure 3B**). A Y-shaped duratomy was then made to expose the lateral superior cerebellar space for endoscopic explorations (10). The tentorium cerebelli was opened for better exposure of the tumor, with attention to CN IV protection (**Figures 3C, D**). The tumor was found to originate from the CN V and remain cephalad to the petrosal vein (**Figure 3D**). Then, the lesion was removed in a piecemeal fashion, with multiple adhesions involving the tentorial notch and cerebellum carefully dissected (**Figure 3E**). After stepwise tumor reduction, the CN III residing in front of the lesion was identified. Importantly, the CN V that was displaced laterally and posteriorly into the plexiform layer was carefully separated from the tumor and preserved as intact as possible (**Figure 3F**).

**Figure 4:**
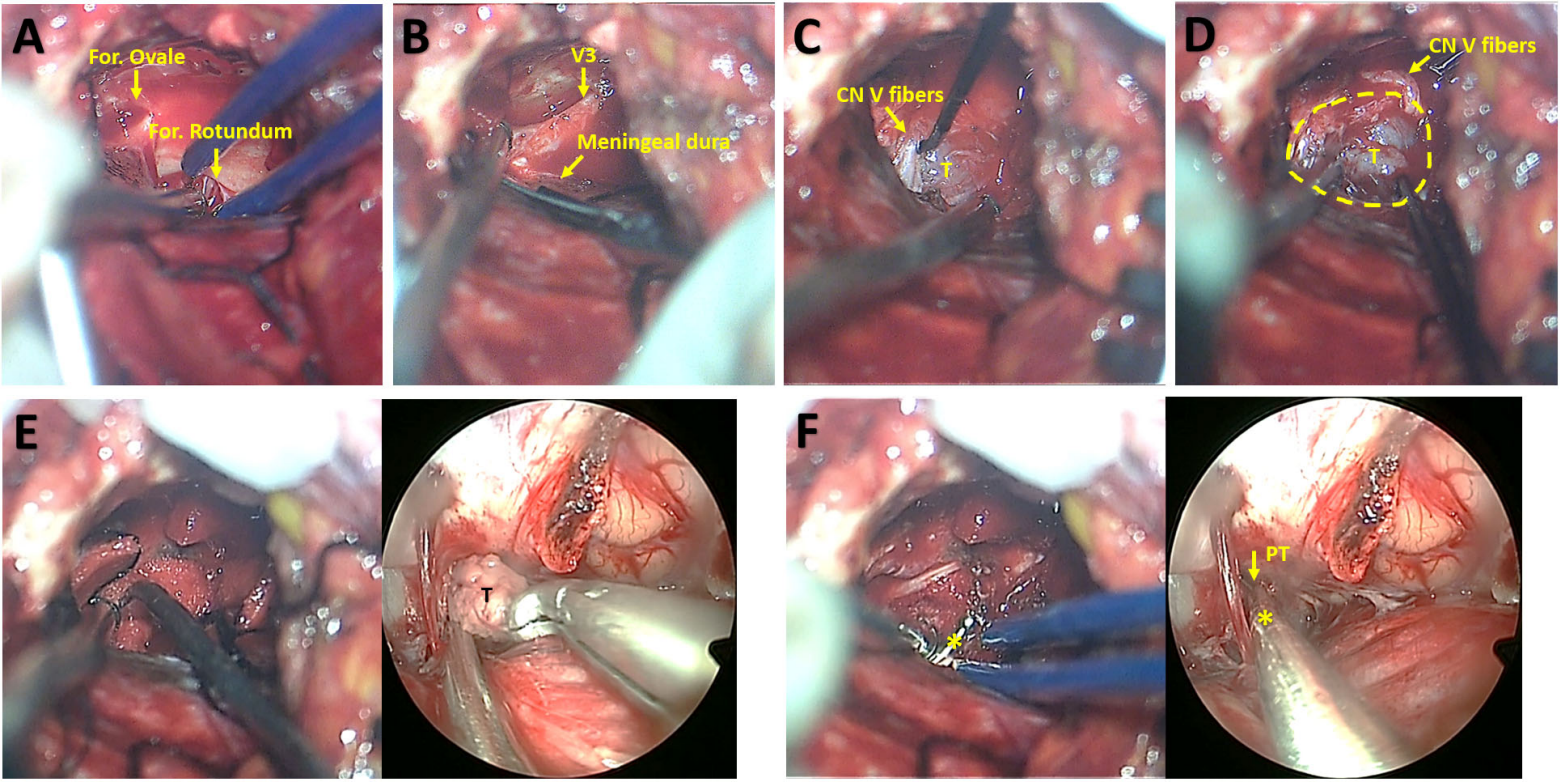
Microsurgical subtemporal-interdural approach for exposure of middle fossa tumor and synchronous microsurgical and endoscopic resection of middle fossa tumor. **(A)** Middle fossa dural peeling toward the lateral edges of the oval and round foramina. **(B)** Detachment the periosteal dura from the meningeal dura of the temporal base for the exposure of Meckel cave. **(C)** Exposure of tumor within the Meckel cave beneath CN V fibers. **(D)** The separation of tumor from the wall of Meckel cave. **(E)** The microsurgical view showing tumor displacement into the porus trigeminus with the aid of cotton-pieces and curette (left) and synchronous endoscopic view indicating tumor extraction *via* the porus trigeminus in the supracerebellar space (right). **(F)** The microsurgical view (left) and synchronous endoscopic view (right) indicating explorations *via* a dissector for residual tumor toward Meckel cave through the porus trigeminus. CN V, trigeminal nerve; For., foramen; PT, porus trigeminus; T, tumor; V3, the mandibular nerve. The yellow circle indicates the boundary of the Meckel Cave. The asterisks indicate the dissector.

A subtemporal-interdural approach *via* temporal craniotomy was performed after endoscopic resection of the posterior fossa segment. Middle fossa dural peeling was performed epidurally to the lateral edges of the oval and round foramina (**Figure 4A**). Then, the periosteal dura was sharply detached from the meningeal dura of the temporal base to expose the MC (**Figure 4B**). The tumor covered with the plexiform portion of the CN V could be exposed by dissecting the fibers on the surface (**Figure 4C**). With gentle movements, the tumor margin was easily separated within the MC (**Figure 4D**). Then, the tumor was pushed into the PT with the aid of cotton-pieces, thus enabling tumor extraction from the supracerebellar space under endoscopy (**Figure 4E**). Finally, endoscopic explorations for residual tumor were permitted toward the MC through the PT under microscopic monitoring (**Figure 4F**).

Postoperatively, the patient had stable trigeminal neuropathy without any new neurological deficits, and imaging examinations indicated that gross total resection was achieved (**Figures 1H–K**). Histopathological examinations confirmed the tumor to be a schwannoma and showed proliferation of spindle cells and strong immunohistochemical staining for S-100 protein.

Institutional Review Board or ethics committee review was not needed since this is a single case report instead of human subject research. The patient consented to publication of his/her image.

## Literature review and discussion

Given the benign nature of TNs, the surgical approach needs to be considered carefully to achieve complete excision with minimal morbidity. Conventional approaches, including frontotemporal transsylvian, subtemporal-intradural, subtemporal-transtentorial, or suboccipital approaches, usually resulted in tumor recurrence exceeding 50% owing to inadequate exposure (11, 12). In the 1990’s, skull-base approaches, including frontotemporal extradural-interdural, frontoorbitozygomatic subtemporal anterior petrosal, and presigmoid posterior petrosal approaches, were introduced. Accumulating evidence demonstrated that compared with conventional approaches, skull-base approaches allowed better exposure, multiple working angles with minimal brain retraction, and higher complete removal rates without increased morbidity (2, 4). Nevertheless, skull-base surgery would by no means dwarf the value of conventional surgical approaches for TN surgery. Conventional approaches are still useful in excising small TNs or decompressing large ones, and play an ancillary but indispensable role in combined approaches or staged procedures that multi-compartmental TNs usually necessitate.

Both frontotemporal and subtemporal craniotomy offered the chance for removal of the middle fossa part interdurally. Zygomatic osteotomy could further minimize brain retraction and allow better access to the anterior temporal fossa (3, 13). Since the expanded MC occasionally seen in TN cases allows entry to the posterior fossa, Al-Mefty et al. advocated posterior fossa tumor removal through the MC. This will be particularly advantageous when venous anatomy prohibits tentorial sectioning or superior petrosal sinus (SPS) coagulation. Since limited exposure of the PT often leads to residual tumor, this natural pathway could be further enlarged by an upward small incision of the PT and even sectioning of the most anterior portion of the SPS (3, 14). Although a certain portion of dumbbell-shaped TNs could be resected in this way, it is not recommended for large posterior fossa component owing to risks of uncontrollable bleeding from the blind corner (1). Instead, tentorial incision or anterior petrosectomy is necessary.

Posterior fossa tumors remaining cephalad to the acoustic-facial bundle could be partially removed by tentorial incision, while anterior petrosectomy further exposes tumors with extensions to the internal auditory meatus (IAC) (4). According to Kawase’ s descriptions, the anterior petrosal approach can access the anterior surface of the ipsilateral pons, the origin of acoustic-facial bundle, CN VI, 1.5 cm of the basilar artery inferior to the trigeminal root (15–17). Under the condition of TNs, this transpetrosal approach better exposes the space inferior to the trigeminal ganglion that often hides the tumor, unlocks the PT, and better exposes the brainstem to which the tumor may adhere comparing with the transtentorial approach. Thus, more neurosurgeons favored a subtemporal extradural-interdural approach with anterior petrosectomy to achieve optimal exposure of dumbbell tumors (2, 4). Although combination with middle fossa interdural and petrosal approaches usually offered radical one-staged resection in most cases, it fails to sufficiently expose tumors extending below the IAC (15, 16).

Combined approach with a posterior route involved should be favorable for large tumors extending below the level of IAC (18). Samii et al. recommended one-stage operation *via* a subtemporal-presigmoid exposure with anterior and posterior (retrolabyrinthine) petrosectomy (19–21). However, the presigmoid approach has inherent shortcomings such hearing loss and operational complexity. Others preferred a combined approach with suboccipital retromastoid craniotomy, either in a one-stage (22) or staged (23, 24) procedures. However, the operator must pass through dense cranial vessels and nerves to access the tumor. In addition, the suprameatal tubercle may obstruct the exposure of the petroclival region from the lateral to medial view.

Accumulating literature reported the efficacy of Gamma knife surgery (GKS) especially with small and medium-sized TNs owing to excellent radiographic tumor control and minimal cranial nerve morbidity (25–27). However, there existed a large proportion of TN patients exhibiting tumor expansion after GKS (25), and some of them still necessitated surgical resection (1, 28). In addition, radiation treatment would pose a huge challenge for total surgical resection and the preservation of cranial nerves. Fukaya et al. recommended that complete tumor removal would offer better psychological effect for younger patients with large-size tumors, even with mild facial hypesthesia after surgery; for asymptomatic or small-sized tumors, GKS therapy could be considered after a wait-and-see strategy (1). Considering these, a surgical resection based on a combined approach was prioritized for maximizing therapeutic benefit in this young patient.

Herein, a small lateral suboccipital craniotomy was adopted to tackle the posterior fossa tumor. Theoretically, this craniotomy provides multiple corridors toward petroclival lesions including the SCITA approach, the retrosigmoid approach, and the suprafloccular transhorizontal-fissure approach, representing “above, below, and lateral” routes, respectively. As shown in this case, the SCITA approach alone is enough to remove the small posterior fossa segment, while the other two are optional for large tumors even with extensions below the IAC. In terms of origin, TNs in the petroclival junction usually grow medial to the cranial nerves V to XII. EF-SCITA supplies the corridor from the medial to cranial nerves and reduces the manipulation of the nerves when compared with the retrosigmoid approach. Additionally, EF-SCITA provides a direct view of the superior-medial part of the MC, a blind spot for the retrosigmoid approach. High-definition wide-angle visualization and close-up observation endowed with endoscopy further facilitate total resection and minimize injury to critical neurovascular structures. Given above, we believe that the EF-SCITA approach, especially when in combination with the retrosigmoid approach, might be an ideal surgical strategy for most posterior fossa TNs.

Recently, EF-SCITA was adopted by Xie et al. to resect petroclival meningiomas with extension to the middle fossa. They observed that EF-SCITA provided exposure of the supratentorial area without any petrosectomy through the tentorial incision, but a small residual was found postoperatively in the anterior part of the middle skull base owing to limited exposure. The exposure of the cavernous sinus by EF-SCITA still needs further anatomical study (9). In addition to the limitations reported, this operation may endangered the internal carotid artery system. Considering these, subtemporal craniotomy was incorporated in this case based on following clinical merits: resection of possible TNs at lateral cavernous sinus *via* transcavernous approach and assistance in resection of tumors in the MC with endoscopy. In this case, middle fossa tumor removal from the posterior direction was facilitated by releasing the tumor in the MC and pushing the tumor from middle fossa side without anterior petrosectomy.

The multi-corridor hybrid technique represents a novel paradigm in the treatment of complex lesions in the suprasellar region (29, 30), ventricular system (31), pineal region (32), and skull base (33, 34), with endoscopic transventricular or endonasal approach, and microscopic transcranial approaches frequently involved. Recently, this concept was introduced by Porto et al. for a near-total resection of complex TNs *via* a combination of orbitozygomatic approach for removing the intracranial portion and endoscopic endonasal and sublabial transmaxillary approaches for resection of sinonasal, infratemporal, and pterygopalatine components (35). Here, we first utilized synchronous endoscopy and microsurgery *via* combined EF-SCITA and subtemporal approach for resection of dumbbell-shaped TN. This hybrid approach has manifold advantages. First, extensive removal of the tumor from different directions is possible in a single operation. Theoretically, this combined approach is sufficient to expose huge dumbbell TNs with extension to the superior orbital fissure anteriorly and to the IAC posteriorly. Secondly, tumor removal can be assisted by pushing the tumor out from the opposite side. Thirdly, compared with anterior petrosectomy, synchronous endoscopy protects CN III and CN IV from a posterior route and avoids potential injury to the SPS.

Despite these merits, we are clearly aware that it is not without flaws. First, this technique necessitates effective collaboration between an experienced skull base expert and neuro-endoscopist. Any desynchronization may increase surgical risks. Secondly, this technique could not avoid inherent shortcomings of subtemporal and suboccipital craniotomy (including damage to temporal lobe, cerebellum, and the cerebrovenous system) and endoscopy (a steep learning curve and difficulties when handling deep bleeding). Thirdly, tentorium incision is still necessitated. However, this is for enlarging supracerebellar space for better exposing the posterior fossa tumor, but not for transtentorial tumor removal as described in subtemporal-transtentorial approach (4) and previous EF-SCITA reports (9, 10). Fourth, there has been several reports regarding one-stage, complete, and safe resection of dumbbell TN through a pretemporal approach (36, 37). It might be a little aggressive for the application of such complex surgery for a moderate size TN in this case. Thus, this hybrid surgery is more suitable for large dumbbell TNs.

## Conclusions

To the best of our knowledge, this is the first case report describing multi-corridor hybrid technique in the treatment of dumbbell-shaped TNs. Overall, this hybrid microscopic-endoscopic surgery is technically feasible and safe, and contributes to maximal resection with less morbidity, which provides neurosurgeons with a viable alternative to traditional approaches to this kind of challenging lesions. Further studies with larger numbers of patients are warranted to confirm the prognostic significance of this technique.

## Data availability statement

The raw data supporting the conclusions of this article will be made available by the authors, without undue reservation.

## Ethics statement

Written informed consent was obtained from the patient for the publication of this case report.

## Author contributions

SF, GL, XL and SH have been involved in the operation and management of the patient. SF and GL designed the report. XL, YB and SH reviewed the literature, drafted the article and prepared the figures. XS, XL and QZ provided important academic inputs during surgery as well as during the revision of this manuscript. All authors contributed to the article and approved the submitted version.

## Supplementary material

Supplementary Video 1 This video shows technical specification of the hybrid surgery for resection of dumbbell-shaped trigeminal schwannoma. Firstly, a purely endoscopic far-lateral supracerebellar-infratentorial approach was performed for removal of poserior fossa tumor. The tumor in the petroclival region was exposed with the aid of tentorium cerebelli incision, with special attention paid to CN IV protection. Then, the tumor was resected in a piecemeal fashion. Along with tumor decompression, the tumor was found to originate from the CN V and compress the nerve into a plexiform layer posteriorly and laterally. Then, the CN V was carefully separated from the tumor as intact as possible. After tumor removal, multiple adhesions involving the tentorial notch and cerebellum were carefully dissected, and then the CN III in front of the tumor could be identified. Secondly, the microsurgical subtemporal-interdural approach was performed to expose the middle fossa components. The Meckel cave was exposed after conventional middle fossa dural peeling. The tumor was found to locate beneath the compressed CN V fibers within the Meckle cave. Then, the tumor margin was carefully separated within the Meckle cave with gentle movements. Finally, synchronous tumor resection was performed *via* combined endoscopy and microscopy. The tumor was pushed into the porus trigeminus with the aid of cotton-pieces by the skull-base neurosurgeon, thus enabling tumor extraction from the supracerebellar space by the neuroendoscopist. After middle fossa tumor removal, endoscopic explorations for residual tumor were permitted toward the Meckle cave through the porus trigeminus under microscopic monitoring.

Supplementary Video
https://www.dropbox.com/s/p9sbgm3xpoqnfz4/Video%201.mp4?dl=0
